# Letter to the editor regarding plastic surgery training in the UK: Results from a national survey of trainee experiences—Reflections for the total workforce

**DOI:** 10.1016/j.jpra.2021.12.003

**Published:** 2022-01-10

**Authors:** Renu Anita Irri

**Affiliations:** Edinburgh Law School, University of Edinburgh, South Bridge, Edinburgh EH8 9YL

*Dear Sir,*

It was with great interest that I reviewed this article focusing on a national survey of trainee experiences.^1^ I consider the effect of moving towards a competency-based training system on the total workforce.

It is a great pleasure to see that trainees are generally more satisfied with their training in comparison to other surgical specialities. However, a proportion of the plastic non-consultant grade workforce train and work outside of the national training scheme, and many trainees gaining experience in ‘non-training’ posts. These positions are necessary to not only facilitate the opportunities for national trainees to achieve indicative numbers within a six-year timeframe but also provide a fully supported service for patients. My concern is that the survey only represents the experience of part of the non-consultant workforce and feelings of stress, lack of autonomy, and burnout could be more prevalent than reported. A move towards a competency-based system can facilitate trainees to complete training in a shorter time frame, for example, through supernumerary training posts.^2^ Nevertheless, the need for service provision would still exist, putting further pressure on the ‘non-training’ workforce. Consequently, the utilisation of supernumerary training posts or similar strategies could lead to further exacerbation of stress or burnout in the ‘non-training’ group, creating dissonance within the workplace or even a two-tiered system. A healthy and cohesive workforce is required to provide optimal service and patient care.

On the other hand, competency-based training can assist persons who take a less than full time route for childcare needs. A path in which a trainee can progress through at their own pace, while also providing greater transparency in what competencies are expected of a trainee, can be an advantage to the less than full time trainee.^3^ Competency-based training could be utilised alongside shared parental leave (SPL) to provide greater flexibility for trainees. SPL can be taken discontinuously over the first year after birth, which could be beneficial for trainees and departments to collaborate to meet trainees needs and service requirements.^4^ A cultural shift towards the acceptance of SPL could alleviate some of the negative feelings felt by trainees and minimise the risk of discrimination.^1^

In addition, specifying competencies can further enable the ‘non-training’ trainee, providing them with the same amount of transparency. The Joint Committee on Surgical Training (JCST) provide a pathway for doctors to apply for the Certificate of Eligibility for Specialist Registration (CESR) who have not completed a GMC approved program.^4^ By providing a competency-based training for the national trainee can aid the ‘non-trainee’ as well and facilitate a more succinct progression through the CESR route.
